# α-Defensin HD5 Inhibits Human Papillomavirus 16 Infection via Capsid Stabilization and Redirection to the Lysosome

**DOI:** 10.1128/mBio.02304-16

**Published:** 2017-01-24

**Authors:** Mayim E. Wiens, Jason G. Smith

**Affiliations:** Department of Microbiology, University of Washington, Seattle, Washington, USA; University of Michigan

## Abstract

α-Defensins are an important class of abundant innate immune effectors that are potently antiviral against a number of nonenveloped viral pathogens; however, a common mechanism to explain their ability to block infection by these unrelated viruses is lacking. We previously found that human defensin 5 (HD5) blocks a critical host-mediated proteolytic processing step required for human papillomavirus (HPV) infection. Here, we show that bypassing the requirement for this cleavage failed to abrogate HD5 inhibition. Instead, HD5 altered HPV trafficking in the cell. In the presence of an inhibitory concentration of HD5, HPV was internalized and reached the early endosome. The internalized capsid became permeable to antibodies and proteases; however, HD5 prevented dissociation of the viral capsid from the genome, reduced viral trafficking to the *trans*-Golgi network, redirected the incoming viral particle to the lysosome, and accelerated the degradation of internalized capsid proteins. This mechanism is equivalent to the mechanism by which HD5 inhibits human adenovirus. Thus, our data support capsid stabilization and redirection to the lysosome during infection as a general antiviral mechanism of α-defensins against nonenveloped viruses.

## IMPORTANCE

Although the antiviral activity of α-defensins against enveloped viruses can be largely explained by interference with receptor binding and fusion, a common mechanism for inhibition of nonenveloped viruses remains elusive. In studies of a prominent human α-defensin that is expressed in the gut and in the male and female genitourinary tract, we discovered striking parallels between the mechanisms of inhibition of HPV and human adenovirus infection. Thus, detailed studies of the impact of α-defensins on the intracellular trafficking of two disparate viruses support a general mechanism of α-defensin antiviral activity against nonenveloped viruses.

## INTRODUCTION

The α-defensins are a class of immune effector peptides with broad antimicrobial activity against bacteria and both enveloped and nonenveloped viruses (1, 2). Conserved biochemical properties of α-defensins, including their net positive charge, amphipathicity, ability to dimerize, and disulfide-stabilized three-dimensional structure, play a role in their antibacterial and antiviral functions (2–9). However, only a limited number of generalized mechanisms have emerged to explain the ability of α-defensins to alter the infectivity of a very broad range of microorganisms. For enveloped viruses, a consistent theme is that α-defensins bind to viral glycoproteins or to cellular receptors and disrupt their ability to mediate attachment and/or fusion (2, 10–12). The interaction of α-defensins with these viral and host proteins can be either glycosylation dependent or independent. Direct evidence for disruption of the lipid envelope by α-defensins, although it has been long postulated as an antiviral mechanism, is lacking. For nonenveloped viruses, we and others have proposed distinct mechanisms based on detailed analyses of specific virus-defensin combinations; however, compelling evidence for a common mechanism has not materialized. For human adenovirus (AdV), α-defensin binding stabilizes the viral capsid against thermal denaturation and mechanical force (13, 14). Consequently, although the virus is able to interact with its receptor and enter cells, it is unable to uncoat in response to host factors in the endosome. This traps the virus in the endosomal system and prevents trafficking of the genome to the nucleus (13, 15). For JC polyomavirus, increased virion stability was also implicated in the altered intracellular trafficking of virus treated with α-defensin (16). However, studies of human papillomavirus (HPV) were confounding. On the one hand, an inability of the viral genome to reach the nucleus was consistent with the phenotypes of AdV and JC virus; however, HPV was shown to uncoat even in the presence of α-defensin (17). Recently, we reported that the α-defensin human defensin 5 (HD5) inhibited host furin cleavage of the minor capsid protein L2 of HPV16 at the cell surface and postulated that blocking this cleavage disrupted L2 functions required for productive infection (18). In this study, we tested a prediction of our finding that furin-cleaved virus (fcHPV16), in which L2 is processed by furin during virus production, would be resistant to HD5; however, we found that fcHPV16 infection is still blocked by HD5. Furthermore, while HD5-treated fcHPV16 entered cells through the endosomal system, HD5 adversely affected dissociation of the major capsid protein L1 and the viral genome. This dissociation event is the critical outcome of viral uncoating that is required for productive infection (19, 20). Thus, HD5 treatment dramatically changed the trafficking of the viral genome and capsid proteins downstream of the early endosome, redirecting them away from the *trans*-Golgi network to the lysosome. In accordance with this, the capsid proteins were degraded faster during infection in the presence of HD5. This mislocalization of the genome to the lysosome accounts for the inhibition of fcHPV16 as well as wild-type HPV16 and mirrors the effect of HD5 on AdV infection, suggesting that capsid stabilization followed by redirection of the genome to the lysosome is a general mechanism of nonenveloped virus inhibition.

## RESULTS

### fcHPV16 PsV is sensitive to HD5.

**FIG 1:**
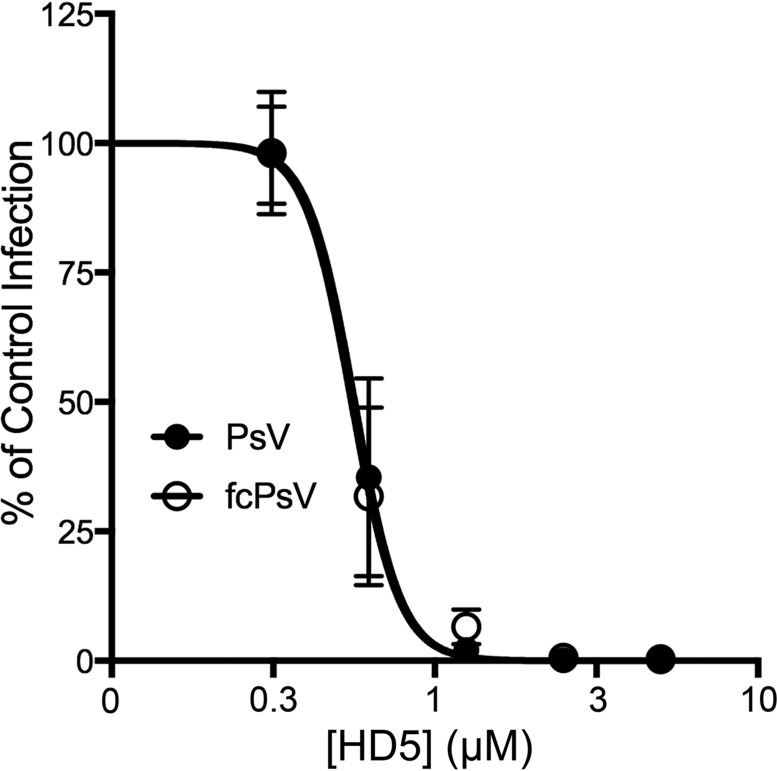
Furin-cleaved HPV16 (fcHPV16) PsV is sensitive to HD5. HeLa cells were infected with uncleaved HPV16 PsV (black circles) or fcHPV16 PsV (open circles) following incubation with increasing concentrations of HD5 in serum-free medium. fcHPV16 samples included 20 µM furin inhibitor. Data from three independent experiments are normalized to infection in the absence of inhibitor ± standard deviation.

To test our prediction that bypassing the need for furin cleavage would preclude HD5 inhibition, we made fcHVP16 pseudovirus (PsV) containing L2 that was cleaved by furin during production (21). Uncleaved HPV16 or fcHPV16 was then incubated with or without increasing concentrations of HD5 and used to infect HeLa cells. HD5 concentrations tested were based on previous studies of HPV inhibition and are within the physiological range of HD5 secreted in the female genitourinary tract (2, 17, 18). Furin inhibitor was added to the fcHPV16 samples to ensure that only precleaved virus would infect cells. Infection was measured by enhanced green fluorescent protein (eGFP) reporter gene expression ~40 h postinfection (Fig. 1). Interestingly, HD5 neutralized both uncleaved and fcHPV16 PsVs at identical 50% inhibitory concentrations (IC_50_s), indicating that while furin cleavage at the cell surface is inhibited by HD5, there is a secondary mechanism by which HD5 blocks HPV16 infection.

### HD5 does not alter trafficking of HPV16 to the early endosome.

**FIG 2:**
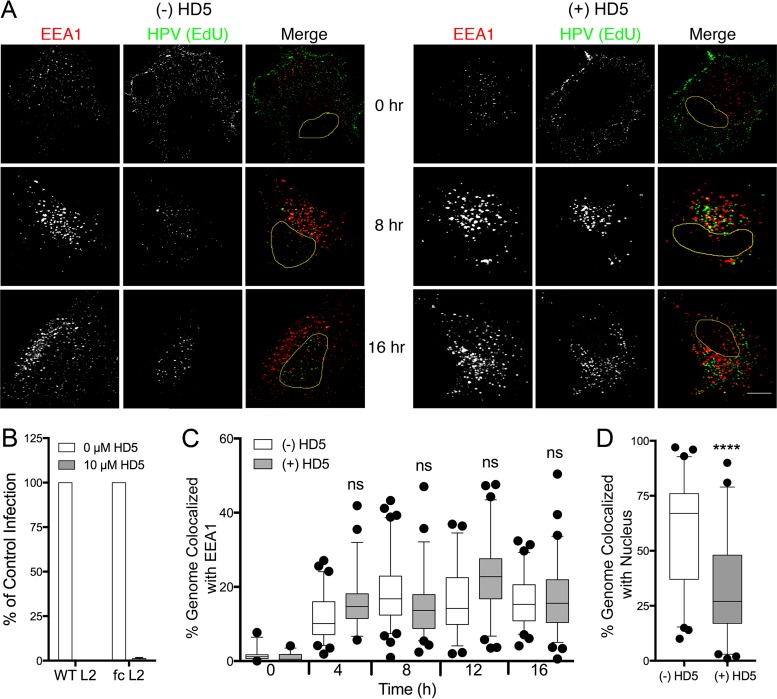
HD5 does not alter fcHPV16 trafficking to the early endosome but blocks the genome from reaching the nucleus. (A) Images of HeLa cells costained for EEA1 and the fcHPV16 EdU genome at the indicated time points postinfection in the presence or absence of HD5. Individual panels depict signal above threshold for images in the z-stack that are coplanar with the nucleus for EEA1 (red) and fcHPV16 genome (green). In the merged images, the nucleus is outlined in yellow. Bar, 10 µm. (B) Infection of HeLa cells by uncleaved HPV16 PsV (WT L2) or fcHPV16 PsV (fc L2) in the presence or absence of 10 µM HD5 in complete medium. Data from three independent experiments are normalized to infection in the absence of inhibitor ± standard deviation. (C) Manders coefficient values M1 (genome colocalized with EEA1) are plotted as a percentage for 40 to 60 cells for each condition in panel A. (D) HD5 blocks nuclear localization of the fcHPV16 genome. The percentage of genome pixels in the nucleus is plotted for 40 to 60 cells from the 16-h-postinfection samples in panel A. (C and D) Whiskers are 5 to 95%, the horizontal line is the median, and outliers are depicted as individual points. ****, *P* < 0.0001. ns, not significant.

After extensive interactions at the cell surface resulting in binding to an unknown internalization receptor, HPV16 enters the cell in a non-clathrin-, non-caveolin-dependent endocytic pathway (22). Previous data have shown that HD5 does not inhibit HPV16 uptake, but the route of internalization was not determined (17, 18). HD5 binding could potentially shunt the virus into an alternative entry pathway, perhaps through an interaction between HD5 and a specific cellular receptor. To explore this possibility, we assessed the colocalization of fcHPV16 PsV, in which the genome was labeled with 5-ethynyl-2′-deoxyuridine (EdU) and EEA1, a marker for the early endosome, at various times postinfection (Fig. 2A). We used fcHPV16 for these studies to exclude the effects of HD5 blocking L2 cleavage. fcHPV16 EdU PsV was prebound to HeLa cells for 1 h at 4°C, unbound PsV was washed off, and cells were further incubated for 1 h at 4°C in the presence or absence of 10 µM HD5. Samples were then shifted to 37°C for 0, 4, 8, 12, or 16 h; stained; and imaged. We first confirmed that 10 µM HD5 neutralized fcHPV16 PsV under these conditions (Fig. 2B). Despite potent neutralization, at no time point did HD5 alter localization of the viral genome to the early endosome (Fig. 2C) (median colocalization in all samples was 10% to 25%). However, the addition of HD5 decreased nuclear localization of the viral genome from ~65% in the untreated sample to ~25% at 16 h postinfection (Fig. 2D). Therefore, although HD5-treated fcHPV16 enters the early endosome with the same kinetics as untreated virus, suggesting that HD5 did not induce viral uptake by an alternative pathway, we confirm the results of Buck et al. (17) that it has decreased nuclear entry and cannot complete infection.

### HD5 inhibits genome dissociation from L1.

**FIG 3:**
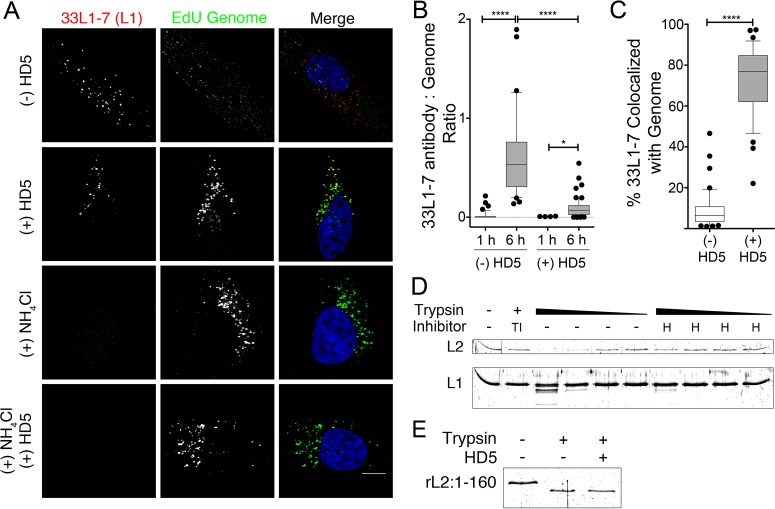
HD5 inhibits L1 and viral genome dissociation by stabilizing the capsid. (A) Images of HeLa cells costained for L1 (33L1-7 antibody) and the fcHPV16 EdU genome at 6 h postinfection in the presence of no inhibitor, 10 µM HD5, 20 µM NH_4_Cl, or both 10 µM HD5 and 20 µM NH_4_Cl. Individual panels depict signal above threshold for images in the z-stack that are coplanar with the nucleus for L1 (red) and fcHPV16 EdU genome (green). In the merged images, the nucleus is blue. Bar, 10 µm. (B) The amount of uncoated L1 is plotted as the ratio of 33L1-7-positive pixels to EdU-positive pixels for 40 to 60 cells for each of the indicated conditions. (C) Manders coefficient values M1 (L1 colocalized with genome) are plotted as a percentage for 40 to 60 cells infected in the presence or absence of HD5 at 6 h postinfection. (B and C) Whiskers are 5 to 95%, the horizontal line is the median, and outliers are depicted as individual points. *, *P* < 0.05; ****, *P* < 0.0001. (D) HD5 protects the HPV16 capsid from trypsin degradation. Purified HPV16 PsV was digested with 4-fold-increasing amounts of trypsin in the presence of trypsin inhibitor (TI), 10 µM HD5 (H), or no inhibitor (-). The highest concentration of trypsin was added to the trypsin inhibitor sample. (E) HD5 does not affect trypsin enzymatic activity. Fifty nanograms of rL2:1-160 was digested with trypsin in the presence or absence of 10 µM HD5. (D and E) Samples were separated via SDS-PAGE, and total protein was visualized using SYPRO Ruby. Gels are representative of three independent experiments.

In the early endosome, HPV uncoating is pH and cyclophilin B dependent (19, 23). Uncoating is a complex process involving multiple conformational changes in the capsid resulting in loss of the barrier provided by the protein shell of the capsid that protects the genome. A previous study found that bromodeoxyuridine (BrdU)-labeled HPV16 was still able to uncoat after treatment with either HD5 or the α-defensin HNP1, using accessibility of the labeled genome to an antibody against BrdU as an indicator of uncoating (17). To confirm this finding, we used an alternative method to measure uncoating based on exposure of the epitope for an HPV L1 antibody (33L1-7) that lies on the internal face of L1 and is accessible only after capsid disruption followed by cathepsin cleavage (19, 24). We also included NH_4_Cl-treated controls to verify that our assay measured only uncoating due to endosomal acidification. HeLa cells were infected as described above with fcHPV16 EdU PsV in the presence of 10 µM HD5, 20 µM NH_4_Cl, both inhibitors, or no inhibitor. Samples were fixed at 1 or 6 h postinfection and stained for immunofluorescence. Because Click-iT chemistry required for EdU staining is known to induce conformational changes in HPV that can result in exposure of the 33L1-7 epitope (19), all samples were stained with 33L1-7 and a fluorescent secondary antibody before EdU staining. As expected, we observed no antibody staining in NH_4_Cl-treated samples or at 1 h postinfection, regardless of the addition of HD5, confirming that exposure of the 33L1-7 epitope is dependent upon endosomal acidification and that addition of HD5 does not overcome this requirement (Fig. 3A and B). To quantify the extent of uncoating in the samples at 6 h postinfection, we calculated the ratio of 33L1-7-positive pixels to EdU-positive pixels. We observed 33L1-7 staining in both the HD5-treated and untreated samples (Fig. 3A and B), although there was less antibody staining in the presence of HD5 than with the untreated control. Collectively, these results are consistent with the previous study (17): HD5 did not mediate an absolute block to viral uncoating as measured by accessibility of an antibody to an internal capsid epitope.

Although our experiments and those of Buck and colleagues (17) demonstrate that even in the presence of HD5, the incoming capsid becomes more permeable and accessible to proteases and antibodies, the critical outcome of uncoating for HPV infection is for L1 and the genome to dissociate (19, 20). Therefore, we reanalyzed our samples for colocalization of the viral genome with L1 that became immunoreactive with 33L1-7, and the effect of HD5 was striking (Fig. 3C). L1 and the HPV genome almost completely dissociated at 6 h postinfection in the absence of HD5 (median colocalization, 5%). However, the small amount of L1 stained with 33L1-7 remained highly colocalized with the genome after HD5 treatment (median colocalization, 75%). Thus, despite reducing but not completely blocking uncoating as measured by changes in the permeability of the capsid, HD5 binding strongly blocks L1 and genome separation that is required for productive infection.

### HD5 binding stabilizes the HPV capsid.

The decrease in 33L1-7 epitope exposure and the inhibition of viral genome and L1 dissociation suggest that HD5 binding might stabilize the capsid. To assess the stability of the HPV16 capsid, we monitored trypsin cleavage of L1 and L2 *in vitro*. Purified HPV16 PsV was incubated with or without 10 µM HD5 on ice for 45 min prior to limited proteolysis by increasing amounts of trypsin at 37°C for 15 min. Samples incubated in the presence of trypsin inhibitor were included as controls, and cleavage of the HPV16 capsid proteins was assessed via SDS-PAGE and SYPRO Ruby staining (Fig. 3D). As expected, the untreated virus was partially digested, while addition of trypsin inhibitor protected both L1 and L2 from degradation. Interestingly, HD5 protected the viral capsid from degradation, as evidenced by retention of full-length L2 and a marked reduction of L1 proteolysis even in the largest amount of trypsin. HD5 does not affect trypsin enzymatic activity directly, since a recombinant L2 peptide comprising the first 160 residues of L2, rL2:1-160, could be cleaved irrespective of the presence of HD5 (Fig. 3E). Taken together with the uncoating data, these experiments suggest that HD5 binds to and stabilizes the capsid in a manner that does not allow for complete uncoating and dissociation of the HPV16 capsid from the genome during entry, a step that is required for viral infection.

### HD5 does not disrupt the normal association of L2 and the viral genome.

**FIG 4:**
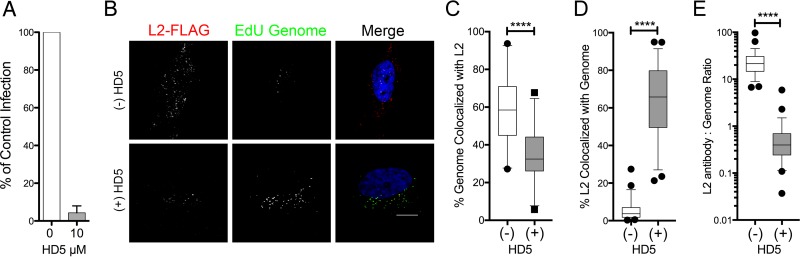
HD5 does not induce dissociation of L2 and the viral genome. (A) Infection of HeLa cells by uncleaved HPV16 L2-FLAG EdU PsV in the presence or absence of 10 µM HD5 in complete medium. Data from three independent experiments are normalized to infection in the absence of inhibitor, ± standard deviation. (B) Images of HeLa cells costained for L2-FLAG and EdU genome at 16 h postinfection in the presence or absence of 10 µM HD5. Individual panels depict signal above threshold for images in the z-stack that are coplanar with the nucleus for L2-FLAG (red) and HPV16 genome (green). In the merged images, the nucleus is blue. Bar, 10 µm. (C to E) Manders coefficient values M2 (genome colocalized with L2) (C), Manders coefficient values M1 (L2 colocalized with genome) (D), and the ratio of the integrated density of the L2-FLAG signal to EdU signal above threshold (E) are plotted as percentages for at least 50 cells each in the absence (-) or presence (+) of HD5. Whiskers are 5 to 95%, the horizontal line is the median, and outliers are depicted as individual points. ****, *P* < 0.0001.

Although endosomal localization of HPV16 was unaltered, the viral genome failed to reach the nucleus after treatment with HD5, indicating that genome trafficking was perturbed. After the virus uncoats, L2 remains bound to the genome and mediates passage of the viral genome to the nucleus (19, 20, 25–27). To assess the interaction of the viral genome and L2, we used uncleaved HPV16 L2-FLAG EdU PsV, a PsV in which the genome is labeled with EdU and L2 is marked with a carboxyl-terminal 3×FLAG tag. We first performed an HD5 neutralization assay to confirm that HPV16 L2-FLAG PsV is sensitive to HD5 (Fig. 4A), since the 3×FLAG epitope is exposed on the capsid surface prior to uncoating (28). Then, HPV16 L2-FLAG EdU PsV was bound to cells for 1 h at 4°C, unbound virus was washed off, and the samples were incubated with or without 10 µM HD5 for an additional hour before shifting to 37°C to allow virus internalization. Samples were fixed at 16 h postinfection and stained for L2 and EdU (Fig. 4B). In untreated samples, the genome was highly colocalized with L2 (median colocalization, 59%) (Fig. 4C); however, reciprocal quantification of L2 with genome was much lower (median colocalization, 5%) (Fig. 4D). This is likely due to the presence of DNA-free (or EdU-free) PsVs. In HD5-treated samples, genome colocalization with L2 was somewhat reduced (median colocalization, 35%) but L2 colocalization with genome was greatly increased (median colocalization, 63%) compared to control. However, we also found that HD5-treated samples had less L2 staining relative to the amount of detectable genome (Fig. 4E). Thus, the quantitative differences in L2 and genome colocalization between HD5-treated and control samples can be explained by reduced L2 staining in the presence of HD5. Nevertheless, we conclude that HD5 does not perturb the normal association of L2 and the viral genome, because almost all of the detectable L2 colocalizes with genome (Fig. 4D). Combined with our previous results showing that HD5 inhibits L1 and genome dissociation, these data suggest that the presence of HD5 maintains a complex of L1, L2, and the genome during cell entry.

### HD5 inhibits trafficking of HPV16 to the *trans*-Golgi network.

**FIG 5:**
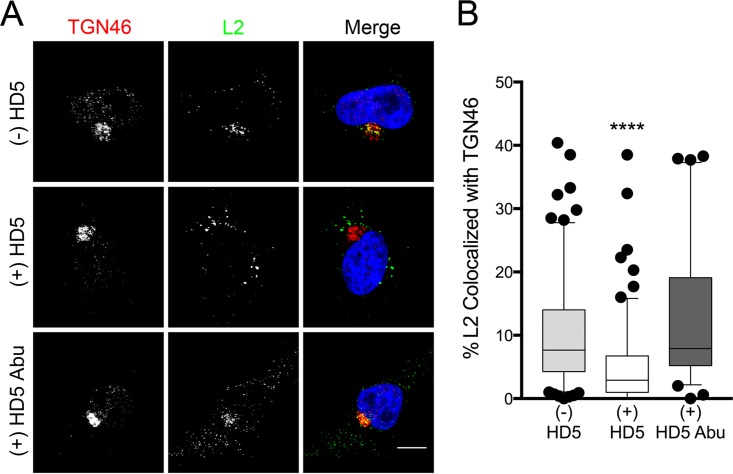
HD5 inhibits trafficking of HPV16 L2 to the *trans*-Golgi network. (A) Images of HeLa cells costained for TGN46 and L2-FLAG at 16 h postinfection in the presence or absence of HD5 or HD5 Abu. Individual panels depict signal above threshold for images in the z-stack that are coplanar with the nucleus for TGN46 (red) and L2-FLAG (green). In the merged images, the nucleus is blue. Bar, 10 µm. (B) Manders coefficient values M1 (L2 colocalized with TGN46) are plotted as a percentage for 40 to 150 cells for each condition. Whiskers are 5 to 95%, the horizontal line is the median, and outliers are depicted as individual points. ****, *P* < 0.0001, comparing HD5-treated cells to either untreated or HD5 Abu-treated cells. All other comparisons are not significant.

Following L1 and L2 genome dissociation, L2 inserts into the endosomal membrane to interact with a cytosolic host protein complex called the retromer (29, 30). The retromer mediates retrograde transport of vesicles containing L2 and the viral genome out of the endosome to the *trans*-Golgi network (20, 28, 29). If the L2-retromer interaction is disrupted, the viral genome cannot reach the *trans*-Golgi network, and the virus is noninfectious (29, 30). To investigate the impact of HD5 on this process, we repeated the immunofluorescence assay using fcHPV16 L2-FLAG PsV to assess the colocalization of HPV16 L2 and the *trans*-Golgi marker TGN46. Note that the 3×FLAG tag prevented complete cleavage of L2, so furin inhibitor was included to block infection of the residual (~15%) uncleaved PsV in the preparation. We also included a linear, nonfunctional HD5 analogue, HD5 Abu, as a negative control (Fig. 5A). In untreated samples and samples treated with HD5 Abu, L2 colocalized with TGN46 (median, 9% and 8%, respectively) at 16 h postinfection (Fig. 5B); however, HD5 treatment reduced the colocalization (median, 4%). Therefore, HD5 treatment inhibits the passage of the viral capsid to the *trans*-Golgi network during infection.

### HD5 directs the viral genome to the lysosome.

**FIG 6:**
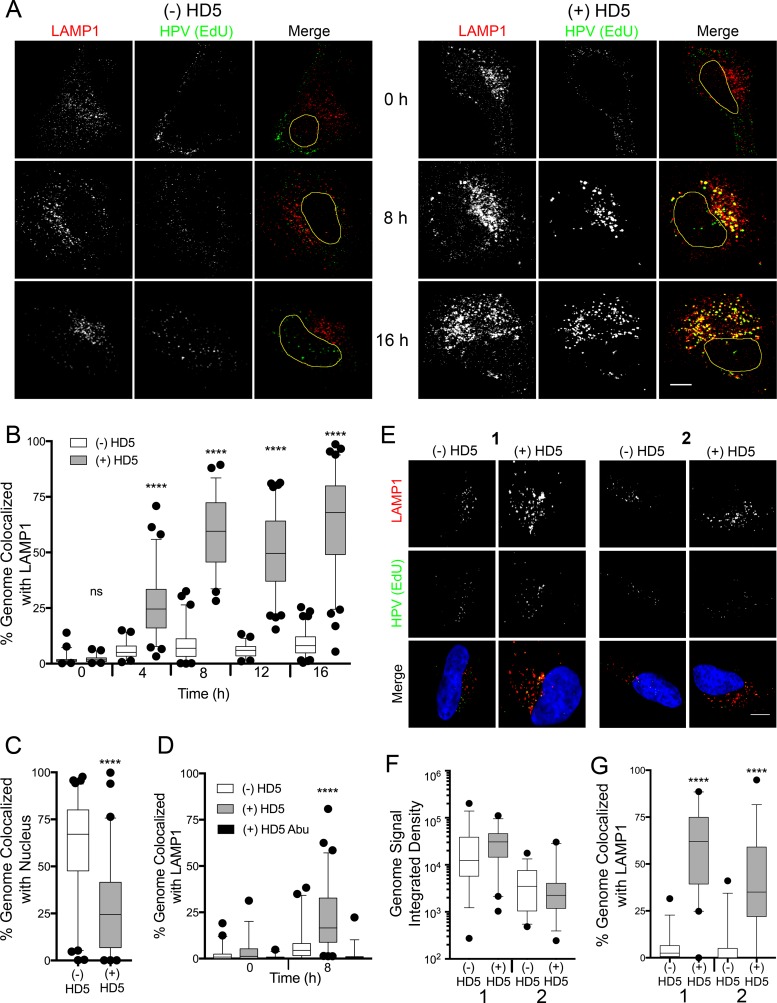
HD5 increases the lysosomal localization of the fcHPV16 genome. (A) Images of HeLa cells costained for LAMP1 and the fcHPV16 EdU genome at the indicated time points postinfection in the presence or absence of HD5. Individual panels depict signal above threshold for images in the z-stack that are coplanar with the nucleus for LAMP1 (red) and fcHPV16 genome (green). In the merged images, the nucleus is outlined in yellow. Bar, 10 µm. (B) Manders coefficient values M1 (genome colocalized with LAMP1) are plotted as a percentage for 40 to 60 HeLa cells for each condition. (C) HD5 blocks nuclear localization of the fcHPV16 genome. The percentage of genome pixels in the nucleus is plotted at 16 h postinfection for 40 to 60 cells. (D) Manders coefficient values M1 (genome colocalized with LAMP1) are plotted as a percentage for 40 to 60 HaCaT cells for each condition. (E) Images of paired HeLa cells costained for LAMP1 and fcHPV16 EdU genome at 8 h postinfection. (F) Integrated density of the EdU signal above threshold for 30 to 40 HeLa cells for each condition. (G) Manders coefficient values M1 (genome colocalized with LAMP1) are plotted as a percentage for 40 to 60 HeLa cells for each condition. For panels B, C, D, F, and G, whiskers are 5 to 95%, the horizontal line is the median, and outliers are depicted as individual points. ****, *P* < 0.0001, comparing HD5-treated cells to untreated cells in panels B, C, and G or HD5-treated cells to untreated and HD5 Abu-treated cells in panel D. All other comparisons are not significant.

We predicted that the viral genome would be transported to the lysosome if HD5 stabilized the capsid and prevented endosomal exit. To test this hypothesis, we infected cells as before with fcHPV16 EdU PsV in the presence and absence of 10 µM HD5 and stained the cells for the lysosomal marker LAMP1 (Fig. 6A). As early as 4 h postinfection, the viral genome in HD5-treated samples was colocalized with the lysosome (median, 25%), and this colocalization increased to 60% at 8 h and later times postinfection (Fig. 6B). In contrast, the lysosomal colocalization of the genome in the untreated samples was negligible (median, 1 to 8%). As in Fig. 2, addition of HD5 reduced the nuclear localization of the viral genome at 16 h postinfection from ~65% to ~25% (Fig. 6C). In both Fig. 2D and 6C, limitations in the resolution of confocal microscopy may explain the residual nuclear localization of the viral genome in the HD5-treated samples despite complete inhibition of infection by HD5.

To confirm the increased lysosomal localization of HPV in the presence of HD5, we repeated the infection in HaCaT cells, a human keratinocyte cell line. We again included HD5 Abu as a negative control (Fig. 6D). At 8 h postinfection, the viral genome in HD5-treated samples colocalized with the lysosome (median, 20%), but not in the HD5 Abu-treated (median, 2%) or untreated (median, 6%) samples (Fig. 6D). The lower levels of colocalization in the HaCaT cells than in the HeLa cells may be due to differences in viral internalization kinetics.

To determine whether or not multiplicity of infection (MOI) has an effect on HD5-dependent intracellular trafficking, we repeated these experiments over a dilution series of input virus. Samples with equivalent levels of total EdU signal per cell at 8 h postinfection (Fig. 6E and F) were then compared for EdU colocalization with LAMP1 (Fig. 6G). Note that in these experiments, as in our previous experiments, HD5-treated samples required a 3- to 6-fold-lower MOI to reach the same amount of internalized virus as untreated controls. We found that in both sets of samples, which differed 7-fold in the amount of internalized PsV, HD5 treatment significantly increased the amount of HPV16 genome that colocalized with the lysosome (median, 65% and 35%) compared to control (median, 2% and 0%). These data support our interpretation that HD5 acts in a virion-autonomous manner to alter trafficking. An alternative model in which HD5 induces aggregation, which we have previously observed when purified HPV16 PsV is incubated with HD5 *in vitro* (18), and aggregation in turn alters the internalization pathway of the virus is not supported. This rationale is further bolstered by our standard experimental design in which the virus is randomly dispersed and prebound to the cell surface prior to the addition of HD5. In sum, HD5 stabilizes the capsid, blocks the release of the L2-genome complex from the L1 capsid shell, presumably prevents L2 interactions with host proteins that are required for endosomal escape, inhibits trafficking of L2 to the *trans-*Golgi network, and ultimately results in accumulation of the genome in the lysosome rather than the nucleus.

### HD5 hastens L1 and L2 degradation in infected cells.

**FIG 7:**
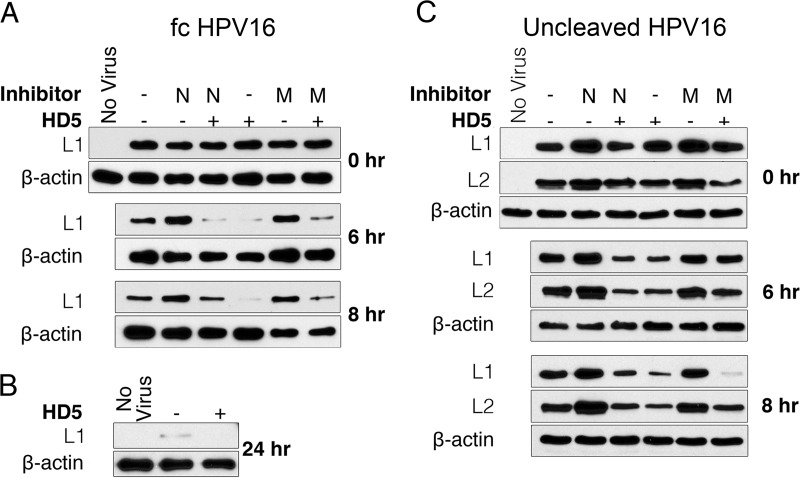
HD5 treatment increases the degradation of fcHPV16 and uncleaved HPV16 capsid proteins. (A and C) Lysates from HeLa cells infected with fcHPV16 (A) or uncleaved HPV Myc-16L2-HA PsV (C) with no inhibitor (-), 20 µM NH_4_Cl (N), or 5 µM MG132 (M) in the presence or absence of 10 µM HD5 were analyzed at the indicated times postinfection. L1 and L2 protein degradation was assessed via immunoblotting. (B) Lysate from cells infected with fcHPV16 in the presence or absence of 10 µM HD5 for 24 h. In all cases, β-actin was used as a loading control, and blots are representative of three independent experiments.

We reasoned that HPV trapped in the lysosome by HD5 would be more rapidly degraded. We therefore assessed the stability of L1 during infection in the presence and absence of HD5. HeLa cells were infected with fcHPV16 EdU PsV; harvested at 0, 6, and 8 h postinfection; and assayed by immunoblotting for L1. The addition of 10 µM HD5 resulted in L1 degradation at both 6 and 8 h postinfection (Fig. 7A). The same extent of L1 degradation was not observed in untreated samples until 24 h postinfection (Fig. 7B). To determine which cellular proteases may be involved in L1 degradation, we tested the effects of NH_4_Cl, which inhibits pH-dependent proteases in the lysosome, and MG132, a proteasome inhibitor. Although both NH_4_Cl and MG132 had some effect on L1 degradation, neither inhibitor prevented the increased L1 degradation caused by HD5, likely due to the fact that multiple proteolytic events contribute to L1 degradation (24). Therefore, increased degradation of L1 is consistent with the altered trafficking of PsVs due to HD5 treatment; however, we were unable to identify the host process responsible.

While much of L1 remains in the endosomal system and is partially degraded even in untreated samples (Fig. 7A), L2 and the genome normally escape the endosome. Since our immunofluorescence data suggest that HD5 treatment inhibits the dissociation of L1 and L2 during infection, we next assessed if HD5 treatment also resulted in increased L2 degradation. We repeated the assay using HPV16 PsV encapsidating an L2 protein with an N-terminal Myc tag and a C-terminal hemagglutinin (HA) tag (HPV16 Myc-16L2-HA PsV). We observed increased degradation of both L1 and L2 in the samples treated with HD5 (Fig. 7C), confirming that the previous observations were not unique to fcHPV16 PsV. Thus, L1 and L2 degradation is consistent with increased trafficking of the HPV16 particle to the lysosome after HD5 treatment. Importantly, we were able to assess genome localization to the lysosome (Fig. 6) at later time points despite this degradative activity, likely because the substrates of the EdU–Click-iT reaction are single nucleotides, allowing the reaction to occur for a period of time after the DNA is partially degraded.

## DISCUSSION

In addition to *Papillomaviridae*, members of three other families of nonenveloped viruses are known to be sensitive to neutralization by α-defensins: *Adenoviridae*, *Polyomaviridae*, and *Parvoviridae* (2). Our current studies, combined with previous work, suggest an overall mechanism for inhibition of HPV infection by HD5 that is generalizable to α-defensin neutralization of these other nonenveloped viruses. First, α-defensins bind directly to the viral capsids rather than acting indirectly on viral infection through cellular targets (8, 13, 16, 18, 31, 32). We have identified a common interface on one facet of the HD5 dimer that is critical for its antiviral activity against AdV and HPV, providing another line of evidence for a conserved mode of action against both viruses (4, 8). Second, the defensin-bound viruses are internalized through their normal cellular receptors and with typical kinetics (13, 16, 32). The only known exception is that BK polyomavirus is aggregated by defensin binding, which prevents binding to the cellular receptor and hence blocks internalization (31). Aggregation of HPV, AdV, and JC polyomavirus occurs but is insufficient to inhibit infection, while the parvovirus adeno-associated virus (AAV) is not aggregated (4, 16, 18, 33). Third, the viral capsids are stabilized upon defensin binding, as demonstrated by resistance to thermal degradation, mechanical force, or proteolysis (13, 14, 16). The net effect of α-defensins on nonenveloped viruses differs from a recently described common mechanism for α-defensin inhibition of bacterial toxins, in which α-defensin binding to disordered regions of these proteins is destabilizing and sensitizes them to proteolysis (34). Fourth, stabilization blocks uncoating in response to host factors during cell entry, and a failure to uncoat precludes key interactions between viral capsid proteins and the endosomal system of the cell that are needed for the genome to traffic to the nucleus. For AdV, the net effect is to prevent the release of the internal capsid protein VI, which normally permeabilizes the endosome and releases the partially uncoated capsid into the cytosol (35). For HPV, HD5 does not completely prevent capsid permeabilization but blocks the usual separation of L1 from L2 and the genome and prevents the trafficking of L2, and presumably the genome, to the *trans*-Golgi network. Interestingly, polyomaviruses traffic from the endosomal system to the endoplasmic reticulum (ER) prior to uncoating (36). This process is also mediated by the capsid, as endocytosed proteins do not generally reach the ER. By reducing ER localization, α-defensin binding has the net effect of preventing JC virus uncoating; however, even virions that reach the ER fail to uncoat, as measured by VP2 exposure (16). Finally, the viral genome accumulates in the lysosome rather than the nucleus. This has been shown directly for AdV and HPV (13), and we predict that this will also be true for AAV and JC polyomavirus. Thus, all of the key elements apply to multiple viruses, supporting the advancement of this paradigm as a general antiviral mechanism for α-defensins.

There remain some unanswered questions regarding the state of the HD5-treated HPV PsV. Defensin binding blocks antibodies from accessing the genome or internal capsid epitopes of AdV but not HPV (15, 17). Thus, for AdV the data support a model in which defensin binding maintains the overall integrity of the internalized capsid (14, 15, 32). For HPV, we cannot distinguish a model in which the capsid is largely intact but permeable to antibodies from one in which the capsid breaks apart but enough L1 remains associated with L2 to prevent L2 interactions with the retromer. Moreover, although we interpret 33L1-7 epitope exposure in the HD5-treated sample as uncoating triggered by endosomal acidification followed by cathepsin degradation, we cannot exclude the possibility that exposure of the epitope occurs exclusively during lysosomal degradation. We assessed uncoating at 6 h postinfection, when untreated HPV16 has reached an acidified endosomal compartment (23). However, a significant amount of the PsV in the HD5-treated sample is already in the lysosome at 6 h postinfection (Fig. 6), and viral proteins are being degraded (Fig. 7). Thus, the HD5 block of HPV uncoating in the endosome may actually be more complete than we surmise.

Overall, our analyses of HD5 inhibition of HPV have revealed two mechanisms that are likely interrelated. Initially, we found that L2 could not be cleaved by host furin in HD5-treated samples (18). Since this was not due to a direct effect of HD5 on furin enzymatic activity, our model is that HD5 binds to the capsid in a way that sterically hinders access of furin to L2. Based on our current data, it is likely that the way in which HD5 interacts with the capsid to block L2 cleavage also precludes the separation of L1 and L2 either by maintaining overall capsid integrity or by stabilizing a subviral complex of L1, L2, and genome. Since HPV capsids isolated from organotypic raft cultures, which may reflect the virion composition of natural infections, are comprised of mixed populations of cleaved and uncleaved virions, multiple neutralization mechanisms may be required for effective defensin antiviral activity *in vivo* (37). The larger question of whether or not the antiviral mechanisms that we have delineated in cell culture occur *in vivo* remains open. Our recent work with mouse AdVs suggests that α-defensins in the small intestine act as adjuvants for enteric viral infection, but it is not yet clear if this is dependent upon altered intracellular trafficking of the virus due to defensin binding (38). Thus, future work to identify where α-defensins bind on the HPV capsid and whether or not α-defensins affect papillomavirus infection *in vivo* is warranted and will likely lead to a better understanding of the molecular mechanisms underlying α-defensin neutralization of diverse pathogens.

## MATERIALS AND METHODS

### Cell culture and pseudovirus production.

293TT cells were a kind gift from Denise Galloway (University of Washington, Seattle, WA). 293TTF cells were a kind gift from Richard Roden (Johns Hopkins University) (21). HeLa cells were purchased from the American Type Culture Collection (ATCC). HaCaT cells were a kind gift from Paul Nghiem (University of Washington, Seattle, WA). Cell culture reagents were purchased from Corning Cellgro. All cells were cultured in Dulbecco’s modified Eagle’s medium (DMEM) containing 10% fetal bovine serum (Sigma-Aldrich), 100 units/ml penicillin, 100 µg/ml streptomycin, 4 mM l-glutamine, and 0.1 mM nonessential amino acids (complete medium). 293TT culture medium was supplemented with 0.4 mg/ml hygromycin B (Sigma-Aldrich). 293TTF culture medium was supplemented with 2 µg/ml puromycin (Sigma-Aldrich).

HPV16 PsV was made via transfection of 293TT cells with plasmids encoding codon-optimized HPV16 L1 and L2 (p16L1L2; kind gift of Martin Müller, GCRC) and an eGFP reporter (pfwB; kind gift of John Schiller, National Cancer Institute, Bethesda, MD). PsV was matured using improved protocols in the lysate of these cells, as described previously (18, 39), and was purified by ultracentrifugation on a discontinuous OptiPrep (Sigma-Aldrich) density gradient (steps of 27% to 33% to 39% OptiPrep). HPV16 Myc-16L2-HA PsV was made by fusing a Myc epitope tag (EQKLISEEDL) to the amino terminus and an HA tag (YPVYDVPDYA) to the carboxyl terminus of L2 via PCR in the p16L1L2 vector. The expression plasmid for HPV16 L2-FLAG, in which a 3×FLAG epitope was fused to the carboxyl terminus of L2 in the p16sheLL vector, was a kind gift from Daniel DiMaio (Yale University) (28, 30). To make HPV16 L2-FLAG PsV, pL2-FLAG was substituted for p16L1L2 during the initial transfection. To make fcHPV16 PsV, we made the following changes to our standard protocol: 293TTF cells were used for the transfection, the lysate was allowed to mature for 48 h, no ammonium sulfate was added to the lysate, and CaCl_2_ was added to the lysate to a final concentration of 5 µM. For EdU labeling, 25 µM EdU was added to the cells 6 h posttransfection. In all cases, the protein content of purified PsVs was quantified by Bio-Rad bicinchoninic acid (BCA) protein assay, which was converted to particle number (3.0 × 10^7^ particles/ng protein).

### Neutralization assays.

Serial dilutions of HPV16 PsVs in serum-free DMEM (SFM) were used to infect HeLa cells in 96-well plates for 4 h. fcHPV16 PsV infection was in the presence or absence of 20 µM furin inhibitor (decanoyl-RVKR-CMK; Calbiochem) to assess the efficacy of the furin cleavage of L2. The cells were washed and cultured in complete medium for ~44 h. Total eGFP expression was quantified with a Typhoon 9400 variable mode imager (GE Healthcare) and ImageJ software (40). A standard curve of infection was constructed in Prism software (version 5.0d; GraphPad Software, Inc.). A virus concentration resulting in ~80% total signal was used in inhibition studies.

To determine antiviral concentrations of HD5, increasing concentrations of HD5 were incubated with HPV16 PsV or fcHPV16 PsV on ice for 45 min in SFM. Folded HD5 was made from a synthesized 80% pure linearized peptide (CPC Scientific, Sunnyvale, CA) and purified by reverse-phase high-pressure liquid chromatography (4). A 20 μM concentration of furin inhibitor was also added to the fcHPV16 PsV samples. The mixture was added to HeLa cells in a 96-well plate and incubated at 37°C for 4 h. Cells were washed and cultured with complete medium for ~44 h. Total eGFP fluorescence was quantified as described above and normalized to a control sample infected without inhibitors using ImageJ. Fifty percent inhibitory concentrations (IC_50_s) were determined using nonlinear regression in Prism.

### Immunofluorescence microscopy.

The day before the infection, 6 × 10^4^ HeLa or HaCaT cells were seeded on glass coverslips. Cells were infected with 1.2 × 10^9^ to 2.4 × 10^9^ particles of fcHPV16 EdU PsV (MOI, ≈2 × 10^4^ particles per cell, assuming a doubling of the cells overnight) or 1.6 × 10^9^ particles of fcHPV16 L2-FLAG. Cells that were to be treated with HD5 were infected with 3- to 6-fold less virus than untreated controls. The virus was incubated with the cells at 4°C to allow binding, unbound virus was washed off with cold medium, and the cells were further incubated at 4°C for an additional hour with medium, 10 µM HD5, or 10 µM HD5 Abu. HD5 Abu is an HD5 analogue containing l-α-aminobutyric acid in place of cysteine, which was chemically synthesized as previously described (32). A 20 μM concentration of furin inhibitor was added to samples infected with fcHPV16 L2-FLAG PsV due to the presence of a minor fraction (<15%) of PsV containing uncleaved L2 in this preparation. Cells were washed with cold phosphate-buffered saline (PBS) and fixed in 2% paraformaldehyde (PFA) for the 0-h time points or shifted to 37°C for the indicated times and then fixed. Cells were permeabilized with 20 mM glycine–0.5% Triton X-100 in PBS for 20 min and then stained with antibody anti-EEA1 (1:250; Cell Signaling), anti-LAMP1 (1:250; Abcam), anti-TGN46 (1:100; Abcam), anti-FLAG (1:1,000; Sigma), or 33L1-7 (1:100; a kind gift from Martin Sapp, LSU Shreveport) diluted in 1% bovine serum albumin (BSA)-0.05% Tween 80-PBS. Secondary antibodies labeled with Alexa Fluor 555 (Life Technologies) were used at 1:1,000. EdU-labeled viral genomes were stained with the Click-iT kit according to the manufacturer’s instructions (Life Technologies). Cell nuclei were stained with TO-PRO-3 (1:1,000; Life Technologies). Coverslips were mounted in ProLong Gold (Life Technologies). z-stack images of least three fields of view for each sample were taken on a Zeiss 510 Meta laser scanning confocal microscope.

Images analysis was performed using ImageJ. Individual cell borders were defined using bright-field images. Only images in the z-stack that were coplanar with the nucleus were used for all image analysis. Background levels of fluorescent stains were determined using uninfected cells. For colocalization studies, Manders coefficients were obtained using the JaCoP plugin of ImageJ for at least 40 individual cells per condition (41). For nuclear colocalization, total EdU-positive pixels in the nucleus were divided by those in the entire cell. For uncoating studies, total EdU-positive and 33L1-7-positive pixels were quantified in ImageJ.

### rL2:1-160 production.

To generate rL2:1-160, an open reading frame (ORF) encoding an N-terminal Myc tag and residues 1 to 160 of HPV16 L2 with a C-terminal 6×His tag was cloned into pRSET-A (Life Technologies). An 0.4 mM concentration of isopropyl-β-d-1-thiogalactopyranoside (IPTG) was used to induce protein expression in BL21 CodonPlus (DE3)-RIPL or BL21-Gold (DE3) *Escherichia coli* (Stratagene), and the protein was purified using Talon resin (Clontech) according to the manufacturer’s instructions. Purified protein was exchanged into 50 mM NaPO_4_-130 mM NaCl via dialysis and stored at −80°C.

### Trypsin cleavage assay.

HPV16 PsV (3 × 10^9^ particles) was diluted in PBS and incubated with or without 10 µM HD5 on ice for 45 min. Trypsin (2.5%; Thermo Fisher) was diluted in PBS and added to samples for final dilutions of 1:2,000, 1:8,000, 1:32,000, and 1:128,000. Samples were incubated at 37°C for 15 min, and SDS loading buffer and dithiothreitol (DTT) were added. Fifty nanograms of rL2:1-160 with or without 10 µM HD5 was incubated with trypsin at a final dilution of 1:2,000 in parallel. Samples were separated on 10% (PsV) or 15% (rL2:1-160) SDS-PAGE gels, and protein was visualized with SYPRO Ruby total protein stain (Thermo Fisher).

### L1 and L2 immunoblotting assays.

HeLa cells were plated in 48-well plates to confluence. Particles (7 × 10^8^) of fcHPV16 EdU or uncleaved HPV16 Myc-16L2-HA were added to cells and allowed to bind at 4°C for 1 h. Unbound virus was removed, and cells were incubated at 4°C for 1 h with or without 10 µM HD5, 20 µM NH_4_Cl, or 5 µM MG132 alone or in combination. Samples were shifted to 37°C for the indicated times, collected by washing with cold PBS, and then lysed with NP-40 buffer (150 mM NaCl, 1% NP-40, 10 mM Tris, pH 8.0) and HALT protease inhibitor (Sigma). Heat-denatured and reduced samples were run on 10% SDS-PAGE gels and transferred to nitrocellulose. Immunoblots were probed for L1 using anti-CamVir (1:1,000; Millipore), for L2 with anti-HA (1:1,000; Thermo Scientific), or using anti-β-actin (1:5,000; Sigma) as a loading control. Anti-mouse secondary antibodies were conjugated to horseradish peroxidase (HRP) (1:5,000; Thermo Fisher), and signal was developed with enhanced chemiluminescence (Bio-Rad).

### Statistical analysis.

For Fig. 2C, 3B, 5B, 6B, and 6D, experiments were analyzed by one-way analysis of variance (ANOVA) with Bonferroni posttests to compare each HD5-treated condition to the control samples or to the control samples and HD5 Abu-treated samples (Fig. 5B and 6D) using Prism. For Fig. 2D, 3C, 4C, 4D, 4E, 6C, 6F, and 6G, experiments were analyzed by unpaired *t* test. For all tests, *P* values of <0.05 were considered significant.
